# Redox‐Sensitive and Bone‐Targeting Self‐Assembled Polymeric Nanomicelles Based on Hyaluronic Acid for Bone Metastasis Treatment

**DOI:** 10.1155/bmri/6081103

**Published:** 2026-06-19

**Authors:** Seyed-Nima Seyed-Mohammadi, Fariba Ganji, Hossein Shaki

**Affiliations:** ^1^ Biomedical Engineering Group, Faculty of Chemical Engineering, Tarbiat Modares University, Tehran, Iran, modares.ac.ir

**Keywords:** alendronate, bone metastasis, hyaluronic acid, nanomicelle, redox-responsive, targeted drug delivery

## Abstract

Treatment methods for bone metastasis often face difficulties because of inadequate blood flow and poor drug absorption in bone tissue. Addressing these challenges, a multifunctional targeting nanomicelle was developed utilizing hyaluronic acid (HA) as the base polymer, functionalized with sodium alendronate (ALN) as a bone‐targeting ligand and an antibone resorption agent (ALN‐HA). To impart a redox‐responsive property, disulfide bonds were integrated into the nanomicelle structure using cystamine (CYS). Finally, vitamin E succinate (VES) was used as the hydrophobic tail of the prepared nanomicelles (ALN‐HA‐CYS‐VES), which have been loaded with curcumin (CUR), as an antitumor agent. The chemical structure of the synthesized polymers was evaluated and confirmed using FTIR and NMR. The mean diameter of prepared nanomicelles was determined as 148.2 ± 2.3 nm, with a narrow distribution size (PDI = 0.169) and critical micelle concentration (CMC) of 49.2 ± 1.8 * μ*g/mL. The drug loading and encapsulation efficiency of CUR were measured as 4.68% and 49.1%, respectively. The drug release profile revealed that approximately 70% of CUR was released in a tumor‐like environment, compared with about 44.7*%* ± 0.9*%* under normal tissue conditions. Hydroxyapatite assay confirmed the high affinity of ALN‐HA‐CYS‐VES for bone mineral matrix (74.20%), whereas this value is only 15.45% for HA‐CYS‐VES. These results indicate that the ALN‐modified HA‐based nanomicelles could emerge as a promising platform for the delivery of anticancer drugs in the treatment of bone metastasis.

## 1. Introduction

Bone metastasis is one of the most challenging types of cancer due to poor blood circulation and inadequate drug absorption in bone tissue, leading to resistance to standard chemotherapy doses. Increasing the dosage is not possible, as it can result in severe toxicity and devastating side effects, hindering the treatment process [1]. Despite all improvements in cancer therapy in the last decades, bone metastasis is still a common and devastating complication for cancer patients, particularly those diagnosed with breast and prostate cancer. It has been estimated that metastatic bone cancer accounts for 90% of prostate cancer deaths and over 70% of deaths from breast cancer [2]. Therefore, the use of intelligent drug delivery systems to combat bone metastasis is crucial.

The microenvironment of bones is highly specialized and provides a suitable platform for the growth and expansion of metastatic cancer [3]. The “vicious cycle” theory was proposed as a means of explaining the rapid growth of metastatic cancer in bones [4]. This theory posits that metastatic cells indirectly cause an increase in the expression of receptor activator of nuclear factor‐kappa B ligand (RANKL) and a decrease in osteoprotegerin expression through osteoblast cells, thereby increasing the number and activity of osteoclasts and increasing bone resorption [5, 6]. Consequently, the increased bone resorption leads to the release of ionized calcium and growth factors, such as transforming growth factor‐beta (TGF‐*β*) and insulin‐like growth factor (IGF), which create an environment conducive to the growth of cancer cells. This creates a cycle that continuously intensifies itself [7].

Bisphosphonate molecules are a family of synthetic drugs with a unique structural similarity to pyrophosphate [8]. The rationale behind bisphosphonates′ affinity for bone tissue is attributed to the oxygen atoms in their phosphate group that exhibit a propensity to form multiple bonds with calcium ions of hydroxyapatite present in the bone mineral matrix [9, 10]. In addition to their bone tissue affinity, these compounds interfere with bone physiology by inhibiting osteoclast activity due to their similarity to pyrophosphates, and it limits the “vicious cycle” of signaling that is further mentioned [11]. Another significant characteristic of bisphosphonates is the fact that their affinity for metastatic bones is 10–20 times higher than for normal bones [12].

Hyaluronic acid (HA) is a biodegradable and biocompatible polymer abundant in the extracellular matrix of mammals. It specifically binds to the “cluster of differentiation 44” (CD44) surface glycoproteins, which are expressed more in cancer cells, such as breast and metastatic breast cancer, bladder cancer, and bone cancer, than in healthy cells [13]. This results in significantly higher endocytosis of HA by these surface receptors in cancer cells than in other cells [14, 15].

Significant research efforts are underway to design stimulus‐responsive drug delivery systems [16]. To design stimulus‐responsive nanocarriers, the selection of an appropriate substance is imperative. This substance should respond to the specific physiological stimulus of the target tissue, thereby preventing abrupt drug release in normal tissues. The concentration of glutathione (GSH) in cellular plasma is approximately two to three orders of magnitude higher than its concentration in the extracellular matrix, which is on the micromolar scale [17]. It has also been reported that the GSH concentration level in tumor tissue is at least threefold higher than in normal tissue. By comparing these values, it can be postulated that the significant difference in the reducing environment can be exploited for specific drug release and differentiation between normal and cancer cells. The tumor‐reducing environment can be utilized as a specific signal for the breakdown of nanocarriers and the release of loaded drugs. Disulfide bonds are easily cleaved by GSH molecules, converting them into sulfhydryl groups that lead to the collapse of the nanocarrier structure and the release of drug contents. Extensive research has been conducted on the use of disulfide bonds for stimulus‐responsive release [9, 18–20].

Herein, we aimed to overcome the multiple barriers to delivering drugs to bone cancer tissue by developing a multifunctionalized HA‐based micellar nanoparticle. HA was utilized as a polymer base. In addition to its nontoxic and biodegradable properties, HA acted as a ligand for CD44 receptors, thereby selectively increasing nanoparticle uptake by cancer cells relative to healthy cells. Sodium alendronate (ALN), as a well‐known bisphosphonate, was conjugated to HA (ALN‐HA) to induce bone tissue–targeting efficacy. Cystamine (CYS), as one of the most common redox‐sensitive compounds, was also incorporated into the nanocarrier structure (ALN‐HA‐CYS). Usually, the CYS disulfide bond breaks down in the presence of GSH and results in rapid drug release in the cancer environment [16]. Finally, vitamin E succinate (VES) was conjugated as the hydrophobic tail (ALN‐HA‐CYS‐VES). Besides its main role in the micellar nanoparticle structure, it can act as an effective drug to reduce angiogenesis activity in tumor tissue and overcome multidrug resistance (MDR) in cancer cells [21, 22]. All conjugates were confirmed by ^1^H NMR and FTIR. Physicochemical properties of prepared nanomicelles, including particle size, polydispersity index (PDI), scanning electron microscopy (SEM), and the critical micelle concentration (CMC), were studied. Curcumin (CUR), a potent antioxidant and anticancer drug, was used as a model drug in this research. The drug loading (DL), drug encapsulation efficiency (EE), in vitro drug release, and bone tissue affinity of CUR‐loaded ALN‐HA‐CYS‐VES (CUR@ALN‐HA‐CYS‐VES) were evaluated.

## 2. Materials and Methods

### 2.1. Materials

HA (Mw = 8 kDa) and ALN were purchased from Arasto Pharmacy Co. (Iran). 1‐Ethyl‐3‐(3‐dimethylaminopropyl)carbodiimide hydrochloride (EDC), N‐hydroxysuccinimide (NHS), and CYS dihydrochloride were purchased from Sigma‐Aldrich Co. (United States). VES was purchased from Exir Co. (Tehran, Iran). CUR, GSH, and pyrene were purchased from Merck (Germany). All other reagents and solvents used in the experiment were of analytical grade and were used without further purification.

### 2.2. Polymer Synthesis

#### 2.2.1. Synthesis of ALN‐HA

ALN‐HA was synthesized following the procedure described elsewhere [14]. To connect ALN to HA, 300 mg of HA (equivalent to 0.75 mmol of glucuronic acid/N‐acetyl‐D‐glucosamine) was dissolved in deionized water. Then, 171 mg of EDC (0.9 mmol) and 103 mg of NHS (0.9 mmol) were added to the solution and stirred on a magnetic stirrer at room temperature for 1 h to activate the carboxylic acid groups of HA. Next, 102 mg of ALN (0.375 mmol) were added to the reaction mixture and stirred at room temperature for 24 h. The obtained product was purified using a 3500 Dalton dialysis bag against deionized water for 72 h. The final product was lyophilized, and the obtained white powder of ALN‐HA was stored at 4°C in a freezer for future use.

#### 2.2.2. Synthesis of ALN‐HA‐CYS

Initially, 100 mg of the ALN‐HA powder was dissolved in 20 mL of deionized water. After adding 151 mg of EDC and 91 mg of NHS, the solution was stirred for 1 h at room temperature to activate the carboxylic acid groups of HA. Then, 102 mg of CYS was added to the mixture and stirred at room temperature for 24 h. The final product, ALN‐HA‐CYS, obtained after 72 h of dialysis against deionized water and freeze‐drying, was stored in the refrigerator for future use.

#### 2.2.3. VES Conjugation to ALN‐HA‐CYS

To conjugate VES, 100 mg of VES was dissolved in 5 mL of dimethylformamide (DMF). The carboxylic acid groups of VES were activated by adding 108 mg of EDC and 65 mg of NHS. Additionally, 50 mg of the ALN‐HA‐CYS was dissolved in 5 mL of formamide, and the VES solution was suddenly added to it. The solution was stirred for 24 h at room temperature. Then, a large amount of very cold acetone was added, and the precipitate was collected using filter paper. The separated precipitates were dissolved in deionized water and dialyzed against deionized water for 24 h to remove excess reactants. After freeze‐drying, the final product, ALN‐HA‐CYS‐VES, was stored in the refrigerator for future use.

#### 2.2.4. Polymer Characterization

The structure of the synthesized polymers was confirmed by the FTIR spectrophotometer (Perkin Elmer Spectrometer Frontier, United States) in the 400–4000 cm^−1^ range. The synthesized polymers were mixed with KBr and pressed into a plate for analysis. The ^1^H NMR spectra were also recorded using an NMR spectrometer (Bruker AV 500 MHz, Germany); D_2_O was used for hydrophilic samples and DMSO‐d6 for amphiphilic samples.

To measure the primary amine content in ALN‐HA‐CYS polymer, a colorimetric assay (TNBS for free amines after conjugation) was employed [23]. The synthesized polymer was dissolved in deionized water (0.25 mg/mL). Then, 600 *μ*L of the solution was mixed with 20 *μ*L of freshly prepared aqueous TNBS solution (1.5 *w*/*v*%) and 200 *μ*L of sodium bicarbonate buffer (NaHCO_3_, 0.1 M, pH = 8.5). The tubes were vortexed for 1 min, followed by incubation for 2 h at 37°C. Then, 600 *μ*L of HCl solution (1.0 N) was added to each tube, vortexed for 30 s, and degassed for 3 min to remove bubbles. The absorbance of samples was measured by the spectrophotometry method at 355 nm (NanoDrop 2000c UV‐Vis spectrophotometer, Thermo Scientific), and primary amine content was calculated according to the calibration curve.

### 2.3. ALN‐HA‐CYS‐VES Nanomicelles

#### 2.3.1. Nanomicelle Preparation

First, 9 mg of ALN‐HA‐CYS‐VES powder was dissolved in 3 mL of DMSO and sonicated to ensure homogeneity. Then, the resulting solution was drawn into a 3 mL syringe and added dropwise to 15 mL of deionized water under vigorous stirring for 2 h using a syringe pump. The resulting solution was transferred to a dialysis bag, and the deionized water in the dialysis vessel was changed after 1, 3, 6, and 12 h. The contents of the dialysis bag were emptied after 24 h.

#### 2.3.2. Nanomicelle Characterization

The average size and PDI of ALN‐HA‐CYS‐VES and HA‐CYS‐VES nanomicelles were determined using the dynamic light scattering (Zetasizer Nano ZS, Malvern Instruments, United Kingdom) method. For this purpose, micelle solutions with a concentration of 0.5 mg/mL were prepared and passed through a microfilter with a pore size of 0.45 mm. Before analysis, the sample microtubes were exposed to an ultrasonic bath to prevent aggregation and clumping in the samples. All measurements were performed at a scattering angle of 90° and a temperature of 25°C with three repetitions. Also, to evaluate the temporal stability of micelles (both blank and CUR‐loaded micelles), in storage conditions, DLS measurements were repeated 1, 2, 3, and 4 weeks after the initial preparation of the samples. SEM (Tescan Mira 2) was also used to observe the morphology of ALN‐HA‐CYS‐VES nanomicelles.

#### 2.3.3. CMC Determination

The CMC was determined by fluorescence spectroscopy using pyrene as a hydrophobic fluorescent probe [24, 25]. In this regard, solutions at predetermined concentrations of ALN‐HA‐CYS‐VES polymer in deionized water were prepared in separate containers. Next, a solution containing 5 *μ*M of pyrene in acetone was prepared. Five hundred microliters of the pyrene solution was added to each of the preprepared polymer solutions. These solutions were left on the stirrer in the dark for 24 h to enable the evaporation of acetone. Then, samples were excited at a wavelength of 335 nm by a fluorimeter (PerkinElmer LS45), and their emission spectra were recorded in the range of 200–800 nm. Pyrene molecules are highly sensitive to light and quickly degrade in the presence of light after the solution is prepared. Therefore, the remaining steps must be carried out in the dark. After recording the emission spectra at different concentrations, the ratio of the intensity of the first peak to the third peak was plotted against the logarithm of the polymer concentration. The CMC value was obtained from the intersection point of the tangent lines drawn on the curve [24].

### 2.4. Preparation of CUR@ALN‐HA‐CYS‐VES Nanomicelles

Different volumes of CUR solution in acetone (2 *μ*g/mL) were added dropwise to the ALN‐HA‐CYS‐VES nanomicelle solution over 1 h (with drug‐to‐polymer mass ratios of 5, 10, 20, and 40). The solution was then placed in an ultrasonic bath for 10 min. The ultrasonic waves stimulated the CUR molecules to move toward the hydrophobic core of the ALN‐HA‐CYS‐VES nanomicelles. The resulting solution was stirred for 2 h and placed under vacuum for 20 min to ensure complete evaporation of the acetone. The loaded nanomicelle solution was then centrifuged at 5000 rpm for 10 min to remove any unloaded CUR. After centrifugation, the remaining solution containing the CUR@ALN‐HA‐CYS‐VES nanomicelles was separated.

#### 2.4.1. DL and EE of CUR@ALN‐HA‐CYS‐VES

The amount of encapsulated drug in the CUR@ALN‐HA‐CYS‐VES nanomicelles was measured through the UV‐visible method. First, 4.5 mL of ethanol was added to 500 *μ*L of the CUR@ALN‐HA‐CYS‐VES nanomicelle solution. The solution was then subjected to ultrasonication for 10 min to break down the micelle structure and release the drug. Afterward, the concentration of CUR in the solution was measured using the UV‐visible at 425 nm (Varian CARY 50 Conc.). The DL and EE of CUR@ALN‐HA‐CYS‐VES nanomicelles were calculated using Equations (1) and (2), respectively.

(1) DL%=encapsulated drugencapsulated drug+polymer×100%

(2) EE%=encapsulated drugadded drug×100%

#### 2.4.2. In Vitro Drug Release Study

As disulfide bonds were integrated into the structure of ALN‐HA‐CYS‐VES nanomicelles to induce a redox‐sensitive release property, the CUR release from nanomicelles was evaluated in the presence and absence of GSH. The physiological GSH concentration in normal tissues is approximately 10–20 *μ*M, whereas in cancerous tissue, its concentration is about a thousand times that of normal body tissues [14]. Therefore, drug release was investigated in an acetate buffer without GSH (to simulate the normal tissue) and an acetate buffer with 10 mM GSH (to simulate the cancerous tissue).

Two milliliters of drug‐loaded nanomicelle solution were placed inside a dialysis bag (MW cut‐off 12 kDa), which was then immersed in 40 mL of buffer solution. The solution was placed in an incubator at a temperature of 37°C and a speed of 100 rpm. The CUR release in each buffer was tested three times. At predetermined time intervals, 3 mL of the release medium was withdrawn to measure the amount of released drug. The withdrawn sample was replaced with 3 mL of fresh buffer. The amount of released drug in the withdrawn sample was measured using UV‐visible analysis (Varian CARY 50 Conc.) at a wavelength of 425 nm. The cumulative amount of the released drug was calculated using Equation (3).

(3) mn=CnVt+∑i=1n−1CiVi

where *m*_*n*_ is the total amount of drug released in the nth stage; *C*_*n*_ is the concentration of the drug, in the *n* ^th^ stage; *V*_*t*_ is the total volume of the release medium; *C*_*i*_ is the drug concentration in the *i* ^th^ sample; and *V*_*i*_ is the volume of the withdrawn sample.

### 2.5. Hydroxyapatite Affinity Assay

To assess the affinity of nanomicelles to the bone mineral matrix, first, 10 mL of CUR@HA‐CYS‐VES and CUR@ALN‐HA‐CYS‐VES micelle solutions were prepared. Then, 100 mg of hydroxyapatite powder was added to each sample, and they were placed in an incubator at a temperature of 37°C, shaking at a speed of 100 rpm for 1 h. The experiments were repeated three times for each solution. Simultaneously, as a control sample, an equal volume of the same solutions without adding hydroxyapatite was placed under the same conditions. After 1 h, the solutions containing hydroxyapatite powder were centrifuged at a speed of 3000 rpm for 15 min, and then the drug concentration in all solutions was measured using the UV‐visible method. Finally, the percentage of binding to hydroxyapatite was calculated using Equation (4).

(4) Binding%=Cwithout hydroxyapatite−Cwith hydroxyapatiteCwithout hydroxyapatite×100%

## 3. Results and Discussion

### 3.1. Polymer Synthesis and Characterization

**Figure 1:**
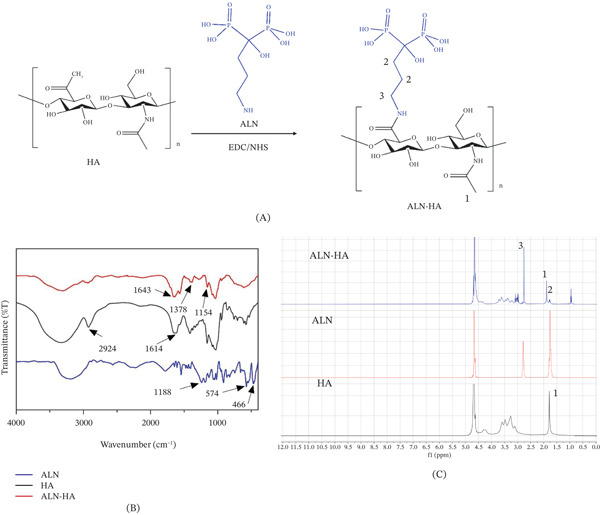
(A) Schematic representation of ALN‐HA synthesis through an amidation process. (B) FTIR spectra of ALN, HA, and ALN‐HA. (C) ^1^H NMR spectra of ALN, HA, and ALN‐HA.

**Figure 2:**
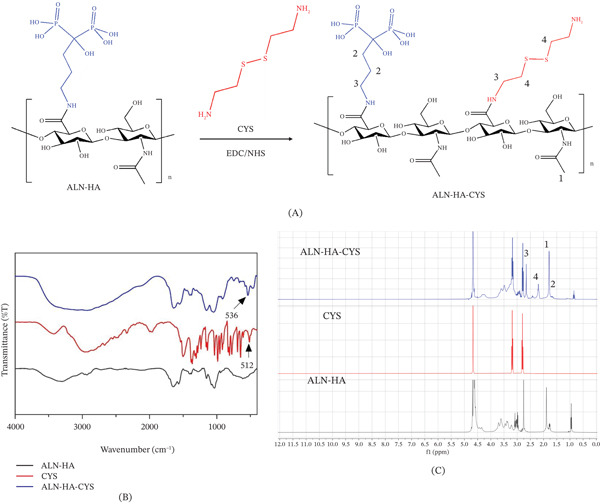
(A) Schematic representation of ALN‐HA‐CYs synthesis through an amidation process. (B) FTIR spectra of ALN‐HA, CYS, and ALN‐HA‐CYS. (C) ^1^H NMR spectra of ALN‐HA, CYS, and ALN‐HA‐CYS.

**Figure 3:**
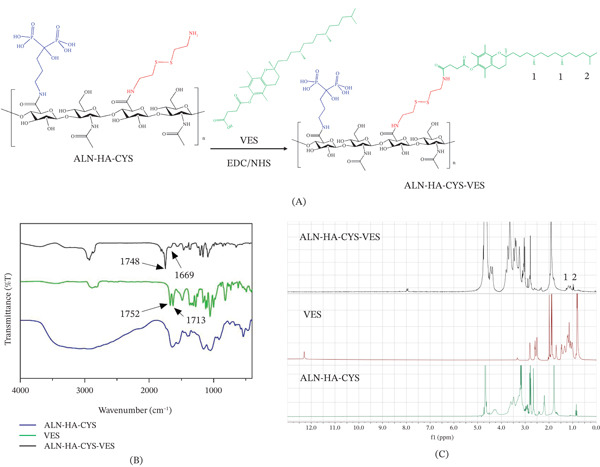
(A) Schematic representation of ALN‐HA‐CYS‐VES synthesis through an amidation process. (B) FTIR spectra of ALN‐HA‐CYS, VES, and ALN‐HA‐CYS‐VES. (C) ^1^H NMR spectra of ALN‐HA‐CYS, VES, and ALN‐HA‐CYS‐VES.

The ALN‐HA‐CYS‐VES polymer was synthesized via a series of amidation reactions (Figures 1A, 2A, and 3A). The chemical structure of the intermediate and the final products was confirmed by FTIR (Figures 1B, 2B, and 3B) and ^1^H NMR (Figures 1C, 2C, and 3C).

In the FTIR spectrum of ALN‐HA, as shown in Figure 1B, absorption peaks at 546 and 579 cm^−1^ correspond to low‐energy vibrations of the phosphonate group, while peaks at 1154 and 1375 cm^−1^ indicate the presence of ALN. The absorption band at 1643 cm^−1^ confirms the successful conjugation of ALN to HA via an amide bond. As shown in Figure 1C, the peak at 1.9 ppm (Peak 1) represents the three protons of the acetamide group in HA. The peak at 2.7 ppm (Peak 3) corresponds to the two protons of the methylene group adjacent to the nitrogen atom in ALN [26]. The cluster of peaks observed at around 1.77 ppm, denoted as Peak 2, represents the four protons of the two remaining methylene groups in the ALN structure [27]. Additionally, two peaks at 4.41 and 4.48 ppm corresponded to methyl protons in the structure of HA. The degree of substitution of ALN in the synthesized polymer, defined as the number of ALN molecules attached per 100 disaccharide units of HA, was calculated by Equation (5):

(5) %DSALN=A2/4A1/3×100%

The degree of substitution of ALN was 30.3% by dividing the area under the peak at 1.77 ppm by the area under the peak at 1.90 ppm (Figure S1).

In the spectrum of ALN‐HA‐CYS, shown in Figure 2B, in addition to the amide bond peak, the peak at 536 cm^−1^ corresponding to low‐energy vibrations of the disulfide bond is also visible, confirming the attachment of CYS. Upon analyzing the ^1^H NMR spectrum of ALN‐HA‐CYS, shown in Figure 2C, it was determined that the peak at 2.72 ppm corresponds to the hydrogen atoms of the methylene group adjacent to the amide bond formed during the amidation reaction. However, this peak represents a combination of amide bonds from both ALN and CYS. The peak at 2.20 ppm (Peak 4) corresponds to the protons of the methylene group adjacent to the disulfide bond in CYS. The degree of substitution of CYS in the ALN‐HA‐CYS polymer, defined as the number of CYS molecules attached per 100 disaccharide units of HA, was calculated by Equation (6):

(6) %DSCYS=A4/4A1/3×100%

Utilizing this equation, the degree of substitution for CYS was determined to be 31.54% by dividing the area under the peak at 2.20 ppm by the area under the peak at 1.90 ppm (Figure S2). To complement the ^1^H NMR analysis, the resulting polymer was also characterized by the TNBS method to determine the primary amine content of CYS. The measured primary amine concentration was 6.5 *μ*mol/g of polymer. This concentration of primary amine in the structure of ALN‐HA‐CYS is found to be nearly 30% grafting (i.e., 30 CYS moieties per 100 repeating disaccharide units of HA), which is in close agreement with the ^1^H NMR results (31.54%).

Finally, as shown in Figure 3B, the spectrum of the amphiphilic polymer derived from the attachment of VES absorption peaks at 2869, 2927, and 2953 cm^−1^ represents symmetric and asymmetric stretching vibrations of methylene and methyl groups present in the final compound. In the ^1^H NMR spectrum of ALN‐HA‐CYS‐VES, formed by bonding vitamin VES, as it is apparent in Figure 3C, a rise in the peak area at 2.74 ppm is observed, representing the hydrogen atoms of the methylene group adjacent to the amide bond. The emergence of new absorption bands within the range of 0.5–1.5 ppm suggests VES′s attachment as a lipophilic molecule and indicates methyl and methylene groups present in its hydrophobic tail.

Although the synthesis process of this amphiphilic polymer involves three amidation steps, the similar procedure in each step simplifies nanoparticle preparation, which is a significant advantage of this method. However, this similarity in polymer component attachments leads to overlapping absorption bands in spectroscopic analyses, making quantitative analysis of these spectra challenging. Moreover, this similarity in attachments has reduced the efficiency of the substitution degree because CYS and ALN molecules compete with each other for attachment to carboxylic acid groups present in HA, resulting in a decrease in their attachment efficiency.

### 3.2. Nanomicelle Characterization

**Figure 4:**
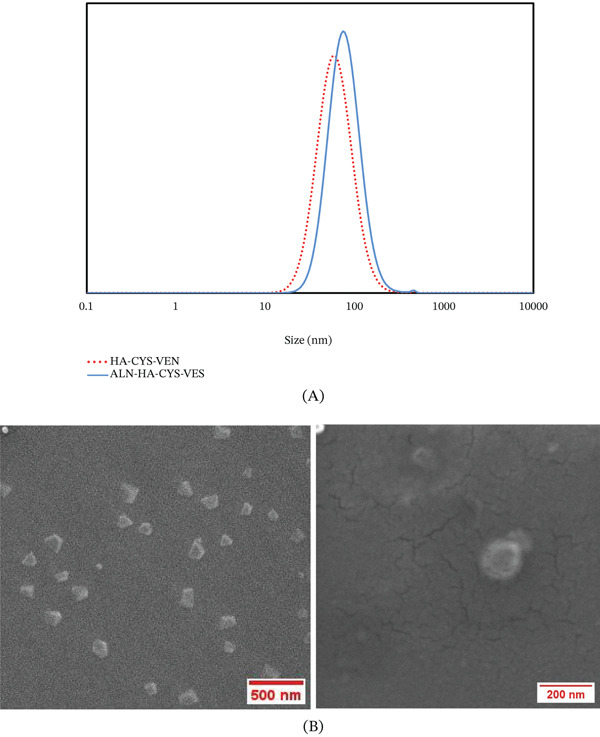
(A) Particle size distribution of the HA‐CYS‐VES and ALN‐HA‐CYS‐VES nanomicelles measured by DLS. (B) SEM images of ALN‐HA‐CYS‐VES.

To investigate the size and size distribution of ALN‐HA‐CYS‐VES and HA‐CYS‐VES nanomicelles, DLS analysis was used (Table 1 and Figure 4A).

**Table 1:** Average size and polydispersity index of micellar nanoparticles.

Polymer	Average size (nm)	PDI
ALN‐HA‐CYS‐VES	148.2 ± 2.3	0.169 ± 0.025
HA‐CYS‐VES	137.5 ± 4.2	0.212 ± 0.041

As shown, nanomicelles containing the bone‐targeting agent ALN display a larger mean particle dimension. Due to its highly hydrophilic nature, ALN reduces the hydrophobic‐to‐hydrophilic ratio of the final polymer, resulting in an increase in nanomicelle size. It is well‐established that as the hydrophobic‐to‐hydrophilic ratio of a polymer increases, the average diameter of the nanomicelles tends to decrease.

The morphology of air‐dried ALN‐HA‐CYS‐VES nanomicelles, as observed by SEM, is shown in Figure 4B. These nanomicelles display a narrow size distribution with an average diameter of approximately 100 nm (using ImageJ software) and a pseudospherical shape. The size discrepancy between DLS measurements (148 nm) and SEM images (100 nm) can be attributed to the differing conditions under which the measurements were taken: SEM captures the dried state of the nanogel, whereas DLS reflects the swollen state. It should be noted that SEM imaging of polymeric nanomicelles is technically challenging due to electron‐beam sensitivity and drying‐induced artifacts. Consequently, DLS serves as the primary method for assessing particle size and size distribution, whereas SEM provides only qualitative morphological support (Figure S3 shows the original high‐resolution SEM image of ALN‐HA‐CYS‐VES nanomicelles to provide a better understanding of particle shape and morphology in the dried state).

### 3.3. CMC of ALN‐HA‐CYS‐VES Polymer

**Figure 5:**
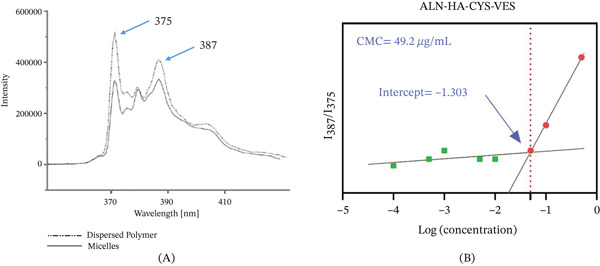
(A) Emission spectra of samples with ALN‐HA‐CYS‐VES polymer concentrations higher and lower than CMC. (B) Plots of intensity ratio *I*_387_/*I*_375_ from excitation spectra versus log C of ALN‐HA‐CYS‐VES.

The fluorescence excitation spectra of ALN‐HA‐CYS‐VES at various polymer concentrations in the presence of pyrene solution are displayed in Figure 5A. The *I*_387_/*I*_375_ ratio plotted against the logarithm of the amphiphilic polymer concentration is shown in Figure 5B. The CMC was determined from the intersection of the two tangent lines fitted to the plotted data. The CMC of the designed ALN‐HA‐CYS‐VES polymer was 49.2 ± 1.8 * μ*g/mL, a value comparable to other similar systems reported in the literature. For instance, Yang et al. [25] synthesized HA‐ss‐FA micelles with a CMC of 36 *μ*g/mL, and Han et al. [28] developed HA‐paclitaxel (PTX) conjugates with a CMC of 43.6 *μ*g/mL. These similar findings underscore the efficiency of amphiphilic polymers in achieving low CMC values, which is a desirable attribute for drug delivery applications. Furthermore, the CMC of the synthesized polymer is significantly lower than that of commercial amphiphilic polymers like Pluronic F127, reported by Lin et al. [29], as 240 *μ*g/mL. This highlights the superior micelle‐forming ability of the designed system compared with commercial amphiphilic polymers, making it a promising candidate for drug delivery with reduced polymer concentration requirements.

The CMC is a crucial parameter for amphiphilic polymers, as it determines the minimum polymer concentration required for the formation of micellar nanoparticles and influences their self‐assembly behavior and stability. A lower CMC is desirable for intravenous drug delivery applications because it reduces the risk of nanoparticle dissociation and premature drug release due to severe dilution during injection, thereby mitigating the risk of drug side effects. It can be hypothesized that an increase in the degree of cysteine substitution and attachment to a constant amount of HA would enhance the structural stability of the micelles and, consequently, lower the CMC.

### 3.4. CUR Loading

CUR was loaded into ALN‐HA‐CYS‐VES nanomicelles at different drug‐to‐polymer ratios. As shown in Table 2, increasing the drug‐to‐polymer ratio leads to a slight increase in CUR loading, but a notable drop in encapsulation efficiency (EE) was observed. This inverse relationship between DL and encapsulation efficiency is commonly observed in nanomicellar systems. DL (DL%) represents the amount of drug successfully encapsulated relative to the total weight of the formulation (drug + polymer), whereas encapsulation efficiency (EE%) represents the percentage of the initially added drug that becomes encapsulated. At higher drug‐to‐polymer ratios, the hydrophobic cores of the micelles become saturated, and additional drug molecules cannot be accommodated within the limited core space. As a result, the excess drug remains unencapsulated and leads to a sharp decline in EE%. Although DL% may continue to increase slightly due to some surface adsorption or deeper packing of drug molecules, the overall encapsulation efficiency decreases because a large fraction of the added drug is lost. This phenomenon can be attributed to the limited space available within the nanomicelle cores. Consequently, as more CUR is added to the primary solution, the greater the drug loss during the encapsulation process. Therefore, an optimal drug‐to‐polymer ratio should be selected, one in which the DL percentage reaches an appropriate level while the EE% also remains at a desirable level. As the results show, increasing the drug‐to‐polymer ratio from 10% to 40% resulted in just a 22% increase in DL% but caused a 79% decrease in EE%. Among the weight ratios of drugs to polymers investigated in this study, the optimal ratio was determined to be 10% *w*/*w* (Table 2).

**Table 2:** Physical characterization of drug‐loaded ALN‐HA‐CYS‐VES nanomicelles.

Drug/polymer ratio	DL%	EE%	Average size (nm)	PDI
10% (*w*/*w*)	4.68 ± 0.22	49.1 ± 2.4	162.6 ± 3.5	0.185 ± 0.021
20% (*w*/*w*)	5.56 ± 0.12	29.4 ± 0.7	172.4 ± 5.2	0.211 ± 0.032
40% (*w*/*w*)	5.73 ± 0.14	15.2 ± 0.5	175.5 ± 4.2	0.205 ± 0.024

The impact of drug‐to‐polymer ratios on DL and encapsulation efficiency (EE) observed in our study is consistent with findings from other researchers working on nanomicellar systems. Xi et al. [15] developed drug delivery systems based on hyaluronan as the backbone and octadecanoic acid as the hydrophobic segment, exploring the effect of varying drug‐to‐polymer ratios on the encapsulation of hydrophobic drugs. They achieved a maximum DL% of approximately 6%, whereas EE% ranged from 8.2% to 37.6%. These values illustrate the challenge of simultaneously optimizing both DL and EE at higher DLs. Similarly, Long et al. [30] employed self‐assembled micelles fabricated from dextrin‐grafted‐octenyl succinic anhydride and cysteamine and reported DL% values for CUR ranging from 0.78% to 2.05%, which are significantly lower than those achieved in our study. These comparisons highlight the enhanced performance of our ALN‐HA‐CYS‐VES nanomicelles, particularly in maintaining higher encapsulation efficiencies while achieving satisfactory DL. The optimal balance of DL and EE identified at a 10% *w*/*w* drug‐to‐polymer ratio in our work indicates an effective strategy to maximize therapeutic payloads without compromising encapsulation stability.

The mean hydrodynamic diameter of CUR‐loaded ALN‐HA‐CYS‐VES nanomicelles was determined by DLS (Table 1). Compared with blank nanomicelles (148 ± 2.3 nm), the CUR‐loaded micelles exhibited a slight but noticeable increase in size. This increase can be attributed to the incorporation of CUR molecules into the hydrophobic core of the micelles. CUR, being a hydrophobic polyphenolic compound, partitions into the core and causes expansion of the core volume, leading to a larger overall hydrodynamic diameter. Similar observations have been reported in other studies where encapsulation of hydrophobic drugs into polymeric micelles resulted in increased particle size [9, 10, 24]. Importantly, the PDI remained below 0.25, indicating that the size distribution remained narrow and that no significant aggregation occurred upon DL.

**Figure 6:**
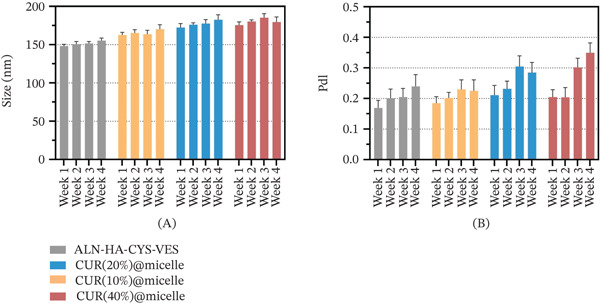
(A) Hydrodynamic diameter size change of blank and CUR‐loaded ALN‐HA‐CYS‐VES nanomicelles over 1 month of storage at 4°C in PBS. (B) Changes in the size distribution of blank and CUR‐loaded micelles over 1 month of storage at 4°C in PBS (mean ± SD, *n* = 3).

It is important to note that although the CMC is a fundamental property of the amphiphilic polymer itself and is typically reported for the blank system, the encapsulation of a hydrophobic drug like CUR can potentially influence the micellization behavior and stability [31–33]. Specifically, the incorporation of drug molecules into the hydrophobic core may alter micellization behavior. The interaction between the hydrophobic drug and the hydrophobic segments of the amphiphilic polymer, which are concentrated in the core of fabricated micelles, can enhance the integrity of the micelles. To directly assess the integrity of the drug‐loaded micelles, we performed long‐term storage stability studies. To evaluate the stability of the blank and CUR‐loaded nanomicelles in storage conditions, DLS measurements were performed immediately after preparation and monitored over a period of 1 month. As shown in Figure 6A, both formulations exhibited a modest increase in their size after a month of storage at 4°C in PBS. The relative stability of the nanomicelles can be attributed to their low CMC value (49.2 *μ*g/mL), which thermodynamically stabilizes the self‐assembled structure and reduces the tendency for dissociation. Additionally, the hydrophilic shell formed by HA and ALN provides steric repulsion, further preventing interparticle aggregation. The moderate increase in size over the 1‐month period is likely due to the high surface‐to‐volume ratio of the nanomicelles, which promotes increased contact among particles and, consequently, some degree of destabilization in aqueous media. As demonstrated by Zhou et al., very small particles are more susceptible to aggregation due to Brownian motion [34]. Nevertheless, the observed size remained below 200 nm for all formulations throughout the study (blank: 155.3 ± 3.2 nm; CUR(10%)@micelles: 170.3 ± 5.7 nm; CUR(20%)@micelles: 182.5 ± 6.5 nm; CUR(40%)@micelles: 179.5 ± 6.7 nm after 1 month). Except for the CUR(40%)@micelles after 1 month (which showed a PDI of 0.350 ± 0.032, above 0.30) (Figure 6B), all PDI values stayed below 0.30, indicating that the nanomicelles remained suitable for drug delivery applications.

Drug leakage from the nanomicelles during storage was assessed by analyzing for free CUR in the solution. No detectable free CUR was found, consistent with CUR′s high hydrophobicity, which keeps it confined to the micellar core. These observations confirm that the DL remained stable, with no significant changes detected throughout storage at 4°C.

### 3.5. In Vitro Drug Release

**Figure 7:**
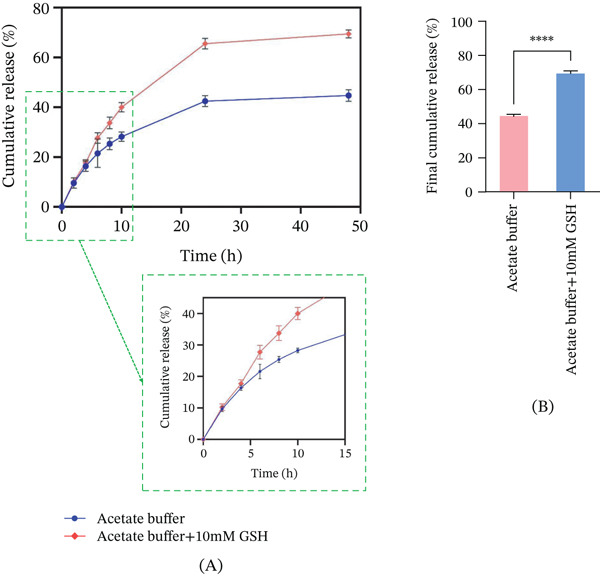
(A) In vitro release behavior of CUR@ALN‐HA‐CYS‐VES nanomicelles in acetate buffer without GSH (•) and acetate buffer with 10 mM GSH (♦). (B) Final cumulative release of CUR from CUR@ALN‐HA‐CYS‐VES nanomicelles in the acetate buffer without GSH and the acetate buffer with 10 mM GSH. Data are presented as the mean ± SD (*n* = 3).  ^∗∗∗∗^Significant differences (*p* < 0.0001).

The release behavior of CUR@ALN‐HA‐CYS‐VES nanomicelles under the aforementioned release conditions is shown in Figure 7A. As it can be seen, the CUR‐loaded nanomicelles exhibited a biphasic release pattern in both media, an initial burst release phase in less than 5 h, followed by a prolonged release phase lasting up to 25 h. The initial burst release of CUR from nanomicelles in the acetate buffer, with or without GSH, is approximately the same (nearly 17%), which could be attributed to the amount of CUR absorbed on the surface of nanomicelles or loosely entrapped between polymer branches. Subsequently, a faster CUR release rate is observed in the release media containing GSH compared with those without GSH. As shown in Figure 7, more than 70% of the loaded CUR was released in the acetate buffer containing 10 mM GSH after 25 h, whereas the total amount of CUR released during the same time in the acetate buffer without GSH is about 44.7*%* ± 0.9*%*. Additionally, Yang et al. reported a 65% release of PTX from PTX/HA‐SS‐VES micelles after 12 h in 10 mM GSH, whereas the PTX released during the same time in the solution without GSH was approximately 46.5% [14]. Ghapanvari et al. reported a 75% release of CUR from an HA‐based carrier [9]. They developed a redox‐responsive carrier with 3,3 ^′^‐dithiodipropionic acid as a redox‐sensitive component. Their finding demonstrated that the drug release pattern was influenced not only by the GSH concentration in the release medium but also by the substitution degree of the redox‐sensitive agent in the polymer backbone of the carrier.

As shown in Figure 7B, these results demonstrated that the presence of a CYS disulfide bond in the nanomicelle structure enabled a responsive behavior to GSH in vitro, representing a good redox sensitivity of ALN‐HA‐CYS‐VES nanomicelles. In the context of stimuli‐responsive drug release, an ideal drug delivery system for cancer therapy would ideally retain the drug under normal physiological conditions and only trigger its release when the carrier nanoparticles reach the cancerous tissue. However, this represents an ideal scenario; in practice, a desirable drug delivery system should minimize drug leakage in normal tissues and selectively release the drug in the cancerous environment. Therefore, drug release from micellar nanoparticles in acetate buffer without GSH was expected to be significantly lower than that in buffer containing 10 mM GSH.

### 3.6. Hydroxyapatite Affinity Assay

Developing a bone‐targeted drug delivery system requires a strong affinity for bone tissue. Given that the microenvironment of bone tissue, especially at the site of occurrence of bone metastasis, is rich in hydroxyapatite, ALN—due to its hydroxyl and phosphonate functional groups and a strong affinity for hydroxyapatite—was employed as a targeting agent in the drug delivery system [12]. To assess the binding capacity of the micelles to bone tissue, an in vitro hydroxyapatite‐binding assay was conducted.

**Figure 8:**
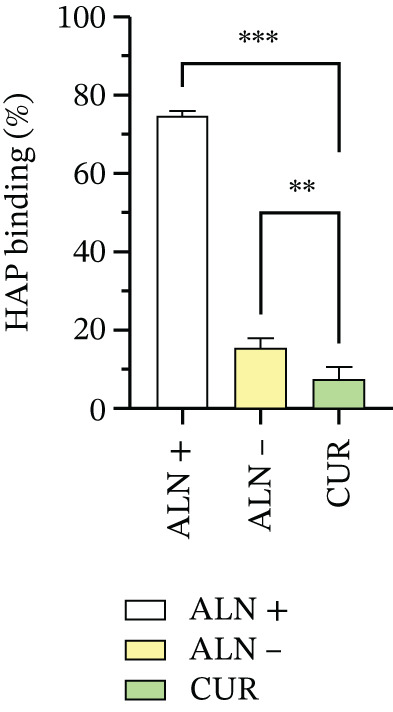
Binding ratio of free CUR, CUR‐loaded ALN‐HA‐CYS‐VES micelles (ALN+), and CUR‐loaded HA‐CYS‐VES micelles (ALN−) with hydroxyapatite. Data are presented as the mean ± SD (*n* = 3).  ^∗∗^ *p* < 0.01 and  ^∗∗∗^ *p* < 0.001.

As depicted in Figure 8, the micellar nanoparticles functionalized with ALN exhibited notably greater affinity to hydroxyapatite compared with the sample without ALN. The binding percentage of ALN‐HA‐CYS‐VES nanomicelles was 74.20*%* ± 3.10*%*, whereas this value was only 15.45*%* ± 2.40*%* for HA‐CYS‐VES nanomicelles and 5.57*%* ± 1.20*%* for the free CUR solution. Similar findings have been reported in other studies, emphasizing the role of ALN in bone‐targeting applications. Ye et al. [7] demonstrated that 85% of DOX@DBMs‐ALN conjugates adsorbed onto hydroxyapatite within 90 min compared with less than 20% adsorption for both free DOX and DOX@DBMs without ALN. The increased binding efficiency observed in our system reflects this parallel trend, highlighting the pivotal role of ALN in enhancing targeted binding to the bone.

Likewise, Xi et al. [15] reported that 79.8% of CUR‐loaded ALN‐HA‐C18 micelles adhered to hydroxyapatite, whereas only 12.5% of free CUR and 17.6% of CUR‐loaded HA‐C18 micelles were adsorbed. These results reinforced the well‐documented affinity of ALN for hydroxyapatite, largely due to its phosphonate and hydroxyl groups, which facilitate strong interactions with bone tissue. Our study corroborates these findings, demonstrating that the incorporation of ALN markedly enhances the binding efficiency of micelles to hydroxyapatite, suggesting that ALN‐functionalized nanomicelles represent a promising strategy for targeting bone metastases.

The results of this test indicated that the degree of ALN substitution on the HA base polymer was adequate, and this amount of ALN functionalization was sufficient for the rapid accumulation of micelles at cancerous bone sites. Alternatively, these results suggest that owing to its water solubility, ALN, as a part of the hydrophilic section of the amphiphilic synthesized biopolymer (ALN‐HA‐CYS‐VES), orients itself outward in the micelle structure. This outward positioning enhances its effectiveness as a targeting agent, promoting improved interaction with the site of action [24].

## 4. Conclusions

A dual‐targeting, redox‐sensitive drug delivery system was engineered to reduce the adverse effects associated with bone metastasis treatment. HA served as a base polymer and CD44‐targeting ligand, ALN was conjugated as a bone‐seeking agent, and VES was connected through a redox‐responsive moiety (CYS), which enabled drug release triggered by the elevated GSH concentration within the tumor microenvironment. In addition, CUR was loaded into the micelles to achieve potential synergistic effects through combination therapy. The polymer′s low CMC (49.2 ± 1.8 * μ*g/mL) supported its potential as a self‐assembled pharmaceutical carrier. This led to the formation of a micellar carrier with an average size below 200 nm and narrow polydispersity, which are crucial characteristics of effective drug delivery systems. Notably, the drug release profile exhibited a markedly accelerated rate under redox conditions mimicking the tumor environment, in contrast to the profile observed under normal tissue conditions. Furthermore, quantitative analysis substantiated and verified the specific affinity of the micelles to the bone mineral matrix via a hydroxyapatite‐binding assay. The present study serves as a proof‐of‐concept focusing on the synthesis, physicochemical characterization, and ex vivo bone‐targeting ability of a redox‐sensitive, bone‐targeting nanomicelle. Although the results are promising, further biological evaluations, including in vitro cytotoxicity assays, cellular uptake studies on CD44‐expressing cell lines, and in vivo biodistribution and efficacy studies in animal models of bone metastasis, are necessary to fully establish the therapeutic potential of this system.

## Author Contributions

**Seyed-Nima Seyed-Mohammadi:** conceptualization, methodology, investigation, data curation, software, validation, writing—original draft, formal analysis, and visualization. **Fariba Ganji:** conceptualization, supervision, resources, project administration, and writing—review and editing. **Hossein Shaki:** project administration, methodology, supervision, and writing—review and editing.

## Funding

No funding was received for this manuscript.

## Conflicts of Interest

The authors declare no conflicts of interest.

## Supporting information


**Supporting Information** Additional supporting information can be found online in the Supporting Information section. The supporting information contains three figures. Figure S1 presents the full‐size ^1^H NMR spectrum of the ALN‐HA polymer with integration values. Figure S2 presents the full‐size ^1^H NMR spectrum of the ALN‐HA‐CYS polymer with integration values. Figure S3 shows the original high‐resolution SEM image of ALN‐HA‐CYS‐VES nanomicelles. These figures are cited in the main text (Sections 3.1 and 3.2) to provide detailed spectral and morphological data.

## Data Availability

Research data are not shared.
