# Supplementary material for: Redox‐Sensitive and Bone‐Targeting Self‐Assembled Polymeric Nanomicelles Based on Hyaluronic Acid for Bone Metastasis Treatment
Source: Biomed Res Int. 2026 Jun 19;2026:6081103. doi: 10.1155/bmri/6081103 (PMC13282557; doi:10.1155/bmri/6081103)
Supplement: Supplementary file 1 — Supporting Information Additional supporting information can be found online in the Supporting Information section. The supporting information contains three figures. Figure S1 presents the full‐size 1H NMR spectrum of the ALN‐HA polymer with integration values. Figure S2 presents the full‐size 1H NMR spectrum of the ALN‐HA‐CYS polymer with integration values. Figure S3 shows the original high‐resolution SEM image of ALN‐HA‐CYS‐VES nanomicelles. These figures are cited in the main text (Sections 3.1 and 3.2) to provide detailed spectral and morphological data. [file BMRI-2026-6081103-s001.docx]

**Redox-Sensitive and Bone-Targeting Self-Assembled Polymeric Nanomicelles Based on Hyaluronic Acid for Bone Metastasis Treatment**

Seyed-Nima Seyed-Mohammadi^1^, Fariba Ganji^[[1]](#footnote-1)^*, Hossein Shaki^1^*

Biomedical Engineering Group, Faculty of Chemical Engineering, Tarbiat Modares University, Tehran, Iran, 14115-143, Iran

**Supplementary**


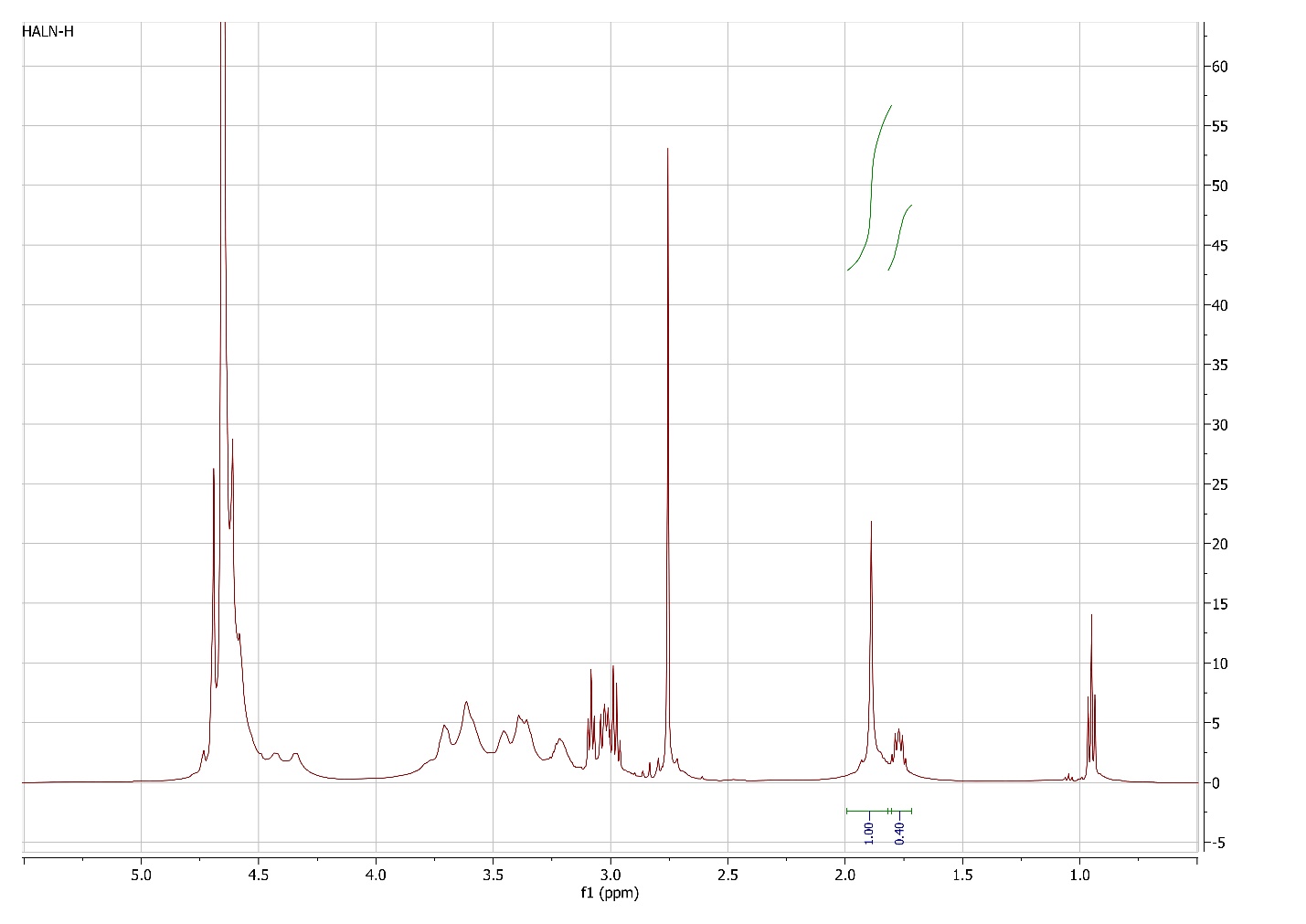


**S1**- ^1^H NMR spectra of ALN-HA.


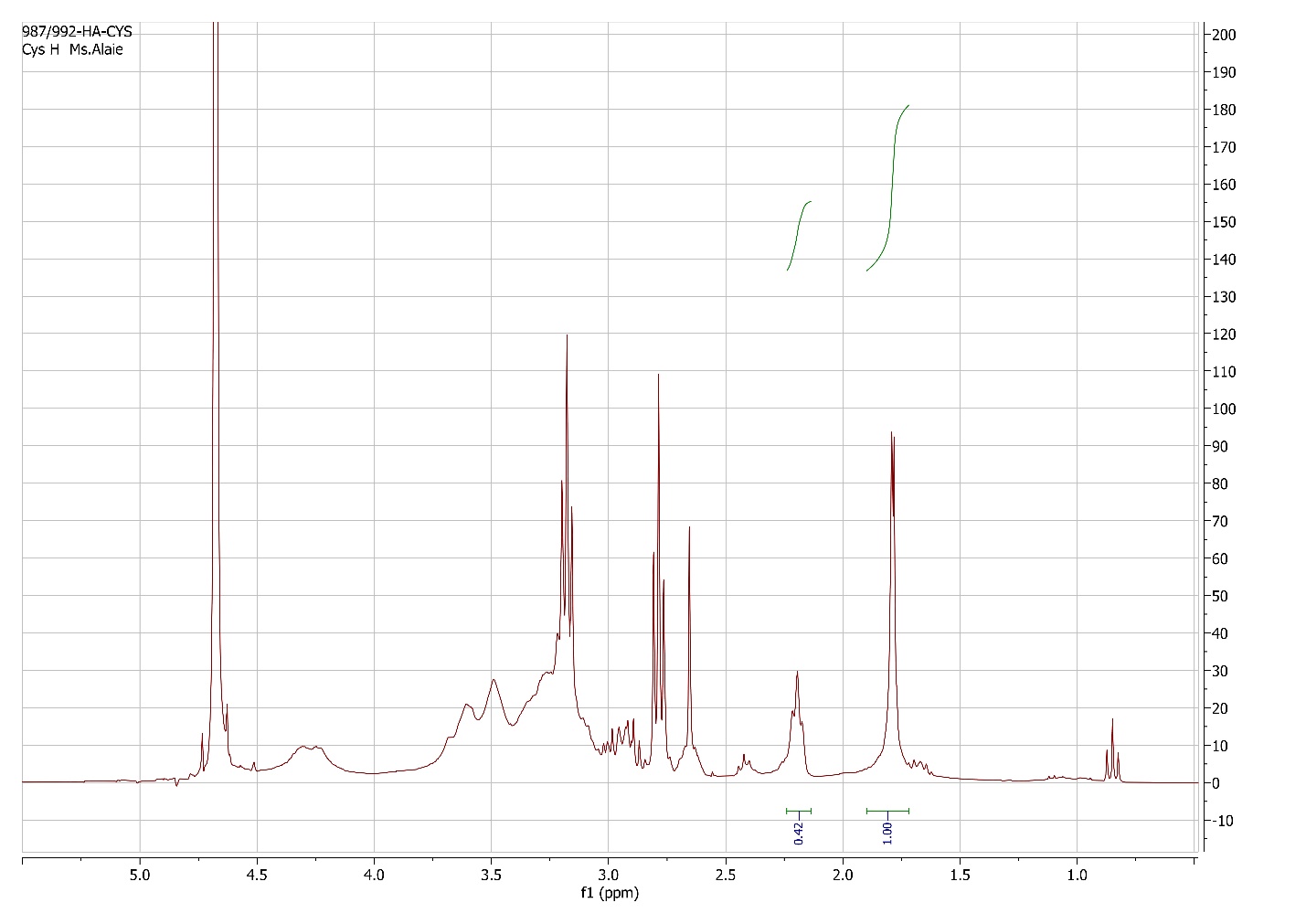


**S2**- ^1^H NMR spectra of ALN-HA-CYS.


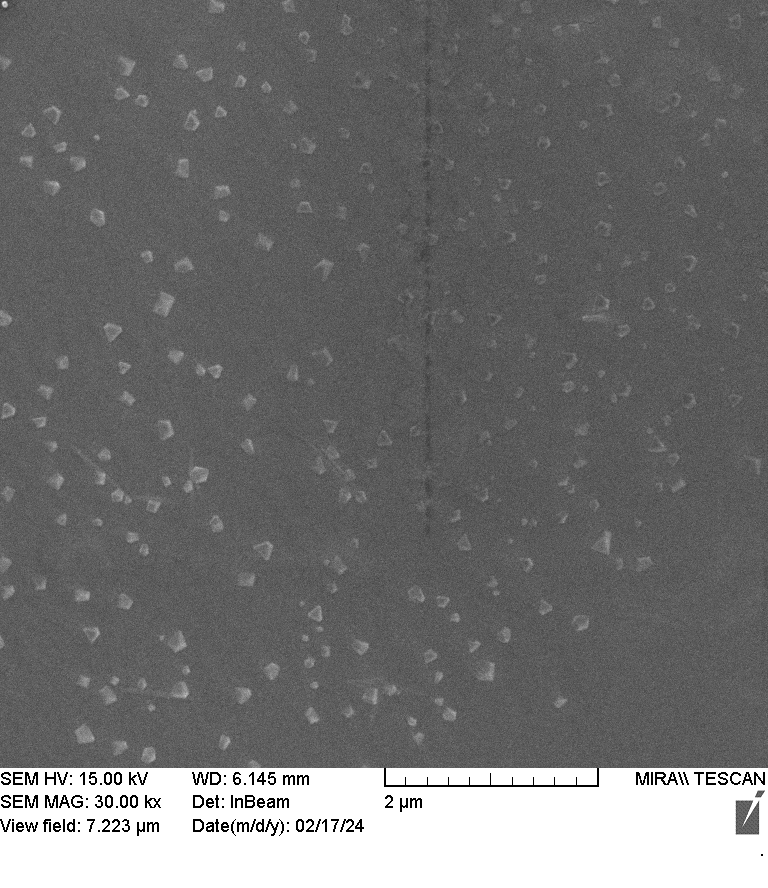


**S3**- SEM images of ALN-HA-CYS-VES

1. Corresponding author: Fariba Ganji, [fganji@modares.ac.ir](mailto:fganji@modares.ac.ir), Hossein Shaki, [h.shaki@modares.ac.ir](mailto:h.shaki@modares.ac.ir) [↑](#footnote-ref-1)
